# Dynamic modeling of uteroplacental blood flow in IUGR indicates vortices and elevated pressure in the intervillous space – a pilot study

**DOI:** 10.1038/srep40771

**Published:** 2017-01-19

**Authors:** Christian J. Roth, Eva Haeussner, Tanja Ruebelmann, Franz v. Koch, Christoph Schmitz, Hans-Georg Frank, Wolfgang A. Wall

**Affiliations:** 1 Technical University of Munich, Institute for Computational Mechanics, 85748 Garching, Germany; 2 Ludwig-Maximilians-University of Munich, Department of Anatomy II, 80336 Munich, Germany; 3 Hospital Dritter Orden, Gynecology and Obstetrics, 80638 Munich, Germany

## Abstract

Ischemic placental disease is a concept that links intrauterine growth retardation (IUGR) and preeclampsia (PE) back to insufficient remodeling of uterine spiral arteries. The rheological consequences of insufficient remodeling of uterine spiral arteries were hypothesized to mediate the considerably later manifestation of obstetric disease. However, the micro-rheology in the intervillous space (IVS) cannot be examined clinically and rheological animal models of the human IVS do not exist. Thus, an *in silico* approach was implemented to provide *in vivo* inaccessible data. The morphology of a spiral artery and the inflow region of the IVS were three-dimensionally reconstructed to provide a morphological stage for the simulations. Advanced high-end supercomputing resources were used to provide blood flow simulations at high spatial resolution. Our simulations revealed turbulent blood flow (high-velocity jets and vortices) combined with elevated blood pressure in the IVS and increased wall shear stress at the villous surface in conjunction with insufficient spiral artery remodeling only. Post-hoc histological analysis of uterine veins showed evidence of increased trophoblast shedding in an IUGR placenta. Our data support that rheological alteration in the IVS is a relevant mechanism linking ischemic placental disease to altered structural integrity and function of the placenta.

Ischemic placental disease is considered to be a unifying pathogenetic concept that connects various obstetric syndromes, particularly intrauterine growth retardation (IUGR) and preeclampsia (PE)^1^, which are the major causes of iatrogenic preterm birth^2^,^3^,^4^,^5^. The concept of ischemic placental disease traces the roots of IUGR and PE back to an early common pathogenetic origin, i.e., insufficient dilation of uterine spiral arteries^6^,^7^,^8^. Dilation of uterine spiral arteries is normally accomplished through remodeling of the arterial media and replacement of endothelium by invading fetal trophoblast cells^9^,^10^ during the first trimester of pregnancy, a phase of predominantly histiotrophic nutrition^7^,^8^. Clinically symptomatic manifestation of the syndromes IUGR and PE occurs beyond the 20th week of gestation^4^,^7^,^11^, after the switch to hematotrophic nutrition.

Those elements of the pathogenetic chain postulated by the concept of ischemic placental disease that range from the early cause to the late onset of symptoms are not fully understood, although the actually proposed mechanisms are rheological by nature^12^. Investigating the structure of the uterine spiral arteries at the microscopic scale and their function *in vivo* is not possible. Furthermore, animal models do not exist in this regard due to substantial species differences in implantation, degree and depth of trophoblast invasion, arterial remodeling and barrier morphology^13^. Blood flow simulations of the uteroplacental circulation are thus a promising way to approach the mechanisms of development of symptomatic diseases beyond the 20th week of gestation. Unfortunately, the resolution limits of ultrasound imaging are also limiting the spatial resolution of such computation models in which both morphological data and flow data were derived from ultrasound examinations^14^,^15^.

A recent review of this problem included static calculations of blood flow through straight tubes with or without conical tube openings (as models of remodeled or unremodeled spiral arteries, respectively), which were based on the Hagen-Poiseuille equation^12^. The calculations performed in the latter study used static flow and pressure conditions and included literature-deduced assumptions of, e.g., uterine artery total flow, numbers of spiral arteries and diameters of arteries with normally dilated and undilated openings. Although this study^12^ indicated the promise of blood flow simulations to explain otherwise inaccessible *in vivo* conditions in human pregnancy, the implemented model still possessed numerous inherent limitations that compromised its proximity to real blood flow conditions *in utero* and thus also its relevance. To achieve the best possible proximity to the *in vivo* situation and to increase relevance, a novel model will have to implement several thus far missing important aspects: (i) rather than straight tubes, spiralized arterial segments similar to those *in vivo* should be included in the model environment; (ii) the intervillous space (IVS) proximal to the arterial opening and the villous tree in the area proximal to the arterial opening have to be included in the calculations; (iii) rheological models will have to exceed the limitations of the Hagen-Poiseuille equation (primarily the restricted validity of the Hagen-Poiseuille equation for laminar flow in tubes only); (iv) modeling algorithms should be able to deliver data on flow velocities, pressure and wall shear stress at vessel walls and at the surface of the villous tree with sufficient spatial resolution in all parts of the model environment, as well as in regions that cannot be considered as tubes (e.g., the IVS or the surface of the villous tree); and (v) the flow model should include flow and pressure fluctuations in the entire model environment during at least one complete maternal heart cycle in the clinically normal, IUGR and IUGR combined with PE (IUGR/PE) conditions.

The model of the present study was designed to meet the aforementioned requirements. It was constructed on a morphological stage with a size of 3000 × 2500 × 2000 μm covering the inflow region of a spiral artery, including a substantial spiralized segment of this artery and the first 2000 μm of IVS and villous tree proximal to the opening of the spiral artery (proximal IVS, see Figure S2 for topology definition). The region was three-dimensionally reconstructed from serial histological sections. The blood flow simulations used an innovative approach with a full description of blood flow properties (velocity profile, pressure profile and wall shear stress profile) at high resolution (550 × 443 × 120 voxels) in the entire morphological stage of the model. Exemplary Doppler flow data of uterine arteries were obtained from a clinically normal patient and from patients with IUGR and IUGR/PE to feed the simulations throughout three full maternal heart cycles.

The present study demonstrates velocity jets projecting into the proximal IVS which correspond to elevated wall shear stress at the villous surfaces in the proximal IVS. Vortices and elevated blood pressure were predicted in the proximal IVS. Circular vortices close to the unremodeled arterial openings are evidence of “dead volumes” of circling blood, which could compromise the efficiency of nutrient exchange. Furthermore, using an innovative, non-routine histological approach, this study also provides evidence that increased wall shear stress can indeed correspond to increased trophoblast shedding from the stressed villous surface.

## Results

### Blood Flow Velocities and Velocity Jets

**Figure 1:**
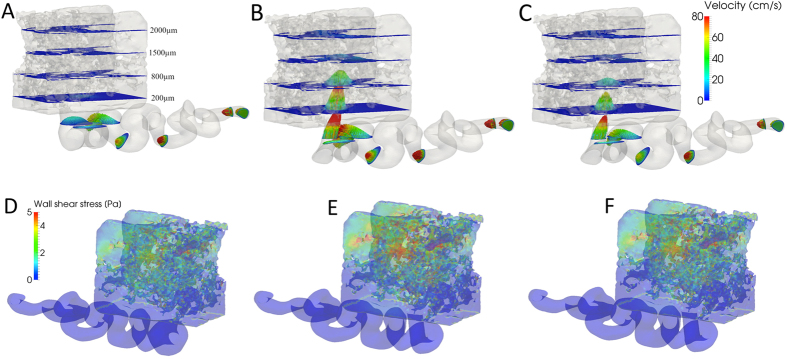
The tiles of the figure each show the entire morphological stage of the model as a light gray background, including the spiralized uterine artery in the lower part and the cuboid block of the proximal IVS in the upper part of each tile. (**A** and **D**) show the model of the clinically normal situation with a dilated arterial opening, (**B** and **E**) the model of the situation in IUGR, and (**C** and **F**) the model of the situation in IUGR/PE. (**A**–**F**) show simulated blood flow at the point of maximum systolic flow of a maternal heart cycle. (**A–C**) show blood velocity vectors, which are color-coded from low (blue) to high (red) velocity; the velocity color scale is shown in C (right to the proximal IVS). The proximal IVS cuboids of (**A**–**C**) contain blue planes that mark the distance from the arterial opening (scale shown in (**A**) right of the proximal IVS). In (**A**) the velocity decreases at the arterial opening, with dominating blue colors at the arterial opening. In (**B**) and less pronounced in (**C**) the velocity accelerates at the arterial opening, forming a velocity jet that projects deeply into the proximal IVS. (**D**–**F**) show wall shear stress; the color scale for (**D**–**F**) (in Pa) is shown in (**D**) (left of the proximal IVS). In the clinically normal situation (**D**), there are moderate to low values of wall shear stress at the villous surface in the proximal IVS. In (**E**) and less pronounced in (**F**) the wall shear stress is elevated in central parts of the proximal IVS, as indicated by more red colors appearing at the villous surface.

The three simulations showed similar flow velocities at the inlets of the arteries, corresponding to the Doppler flow data. In the clinically normal situation, the velocity decreased at the arterial opening and the blood flow remained slow throughout the proximal IVS (Fig. 1A). The pathological cases (Fig. 1B,C), however, showed high-velocity jets in the entry region into the proximal IVS and a snap-through into the more distant regions. Jet streams started after 0.55 sec of the pulse cycle at a velocity of 70 cm/s and showed peak velocities that were a factor of 5 higher in IUGR and a factor of 4 higher in IUGR/PE compared to the clinically normal situation.

### Wall Shear Stress

The computed wall shear stresses at the surfaces of the villous trees in the proximal IVS were low in the model of clinically normal pregnancy (Fig. 1D). In contrast, both pathological conditions showed elevated levels of wall shear stress at the surfaces of the villous trees in the proximal IVS, which were slightly more pronounced in IUGR than in IUGR/PE at an intermediate distance from the arterial opening (Fig. 1E,F). The maximum values for computed wall shear stresses are listed in detail in Table S2, and the rise of wall shear stress during the maternal heart cycle is shown in the movies (Movie S1a for clinically normal and Movie S1b for IUGR) in Supplementary Information.

### Turbulent Flow and Vortices

**Figure 2:**
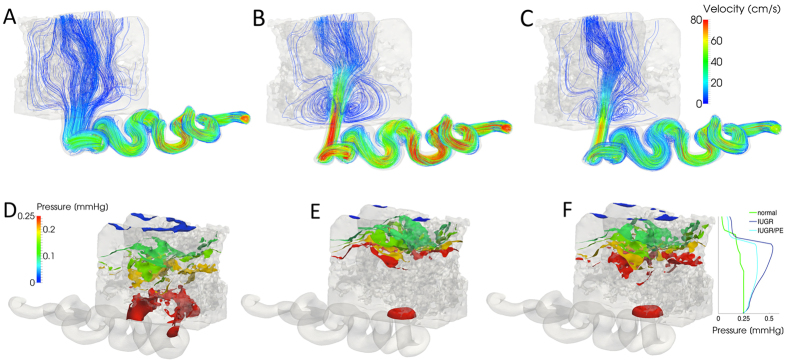
The tiles of the figure each show the entire morphological stage of the models as a light gray background, including the spiralized uterine artery in the lower part and the cuboid block of the proximal IVS in the upper part in each tile. (**A**–**F**) show the situation in the models at the point of maximum systolic flow of a maternal heart cycle. (**A** and **D**) show the model of the clinically normal situation with a dilated arterial opening, (**B** and **E**) show the model of the situation in IUGR, and (**C** and **F**) show the model of the situation in IUGR/PE. In (**A**–**C**) streamlines visualize the course of individual erythrocytes with the velocity color coded along the streamlines; the velocity color scale is shown in (**C**) (right of the proximal IVS). In (**A**) the streamlines run smoothly without turbulence at low speed from the arterial opening into the proximal IVS. In (**B**) and less pronounced in (**C**) the streamlines show the development of vortices in immediate proximity of the arterial opening. (**D**–**F**) show color-coded iso-pressure surfaces, which are spaced by an interval of 0.05 mm Hg; the color scale for (**D**–**F**) (in mm Hg) is shown in (**D**) (left of the proximal IVS). In the clinically normal situation (**D**), the pressure decreases evenly through the proximal IVS toward the upper boundary (at the upper boundary, the pressure is defined as zero). In E and less pronounced in (**F**) the pressure remains elevated in a large part of the proximal IVS and then rapidly drops toward the upper boundary by compressing the iso-pressure surfaces at a short distance. The insert right of the proximal IVS in (**F**) visualizes the course of pressure along individual streamlines between the arterial opening and upper boundary for all three models.

Streamlines were used to visualize directional flow patterns; they visualize the course of single erythrocytes in the model (Fig. 2A–C). In the clinically normal situation (Fig. 2A), the flow widened without turbulence from the arterial opening into the proximal IVS. In the pathological situations (Fig. 2B,C) - more pronounced in the IUGR simulation - the streamlines demonstrated the occurrence of a vortex, i.e., a recirculation zone close to the opening of the uterine spiral artery. In the clinically normal situation the number of streamlines in the arterial opening corresponded to 100% and, thus, 100% of the streamlines reached the top of the proximal IVS. In contrast, only 82.4% (IUGR) and 86.3% (IUGR/PE) of the streamlines reached the top of the proximal IVS in the pathological conditions, while 17.6% (IUGR) and 13.7% (IUGR/PE) of the streamlines shunted into the vortex.

Along a maternal heart cycle, the flow in the entry region into the proximal IVS of the simulated IUGR placenta sample remained laminar over a large time span and only became turbulent once a critical value of flow (Q^∗^ = 0.035 ml/s) was exceeded. Below this threshold, the blood flow patterns were similar to those of the clinically normal situation. The threshold triggered appearance of the vortex in IUGR is shown in a movie in Supplementary Information (Movie S2).

### Blood Pressure Profiles

The total pressure drop over the entire model, i.e., between the artery inlet and the reference point (pressure defined as p = 0 mm Hg) at the top of the proximal IVS was ∆p = 80 mmHg in the clinically normal situation and 1.12 (IUGR) to 1.53 (IUGR/PE) times higher in the pathological cases (Table S2). This difference in pressure profiles could be subdivided into two general phenomena: (i) pressure drop along the spiralized arterial segments including the opening and (ii) pressure drop in the proximal IVS (see Figure S2 for definition).

The majority of the pressure drop occured along the spiralized artery and the arterial opening into the proximal IVS. The pressure drop in the arterial opening was 20 times steeper in IUGR and 14 times steeper in IUGR/PE compared to the clinically normal situation (Fig. 2D–F, Table S1). The pressure drop in the arterial opening along a maternal heart cycle is presented in Supplementary Information (Movie S3a for the clinically normal situation and Movie S3b for IUGR).

At the entry into the proximal IVS, the pressure dropped to values below 1 mm Hg. Iso-pressure surfaces were used to visualize the pressure in the proximal IVS; these surfaces connect points of the models with identical pressure. In the clinically normal situation, the iso-pressure surfaces were evenly distributed along the flow path through the proximal IVS and indicated a gradual and smooth decrease of pressure (Fig. 2D). Following the course of pressure along individual streamlines (Fig. 2, insert right of F), the pressure smoothly dropped throughout the proximal IVS in the clinically normal situation. In both pathological models, the pressure dropped biphasically, no longer gradually and continuously (Fig. 2E,F). In a substantial part of the proximal IVS close to the arterial opening, the pressure increased to high levels, which were elevated by a factor of 2.16 (IUGR) and 1.58 (IUGR/PE) compared to the clinically normal situation. Close to the upper boundary of the model, the pressure steeply dropped to reach the preset zero reference pressure at the upper model boundary, thereby compressing the iso-pressure surfaces at a short distance. This result indicates substantially higher pressure values at the middle and bottom of the proximal IVS compared to the clinically normal situation.

### Total Volume Flow in the Models

**Figure 3:**
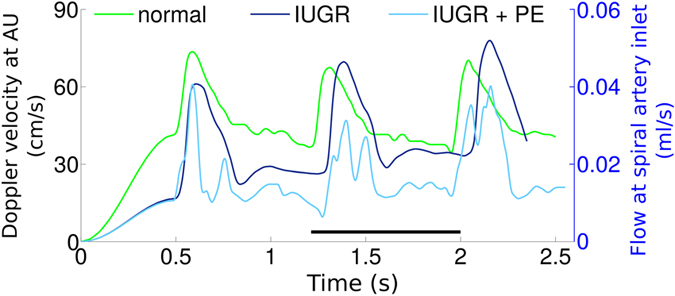
The figure shows Doppler ultrasound waveforms over time measured at the uterine artery from clinically normal (normal; green), intrauterine growth restriction (IUGR; blue), and intrauterine growth restriction with preeclampsia (IUGR/PE; light blue) pregnancies. Time is shown on the x-axis, Doppler flow velocity in the uterine artery is shown on the left y-axis, and flow at the spiral artery inlet is shown on the right y-axis. Flow velocity in cm/s is the original output of the ultrasound analysis; flow velocity in ml/s is calculated from the original ultrasound data using the diameter of the respective arteria uterina. The entire waveforms were used to feed the dynamic flow model of the present study, and the interval of 1.2 to 2.0 s (indicated by the black bar in the upper panel) was used for extraction of the evaluation data.

Doppler ultrasound from clinically normal, IUGR and IUGR/PE pregnancies were exemplary and typical waveforms for the investigated clinical syndromes (Fig. 3 and Table 1). The total blood flow delivered through both uterine arteries was in the range of 339–593 ml/min and in good agreement with previously reported estimates^16^,^17^. For all further calculations, the lower limit of 339 ml/min was chosen to assume reasonable but minimally provocative conditions with respect to turbulence formation. The mean blood flow in a single spiral artery over one full maternal heart cycle equaled Q = 0.031 ml/s in the clinically normal situation, Q = 0.027 ml/s in IUGR and Q = 0.017 ml/s in IUGR/PE pregnancies (Table S2). The maximum flow value during maternal systole was similar in the three cases (Fig. 3). However, the flow was unstable and exhibited notching in the IUGR/PE case, which reduced the average blood flow per heart cycle. Consequently, the IUGR/PE case showed reduced vortex build-up as the critical flow value Q was only briefly exceeded during systole.

### Post-hoc Histological Exploration of Shedding

**Figure 4:**
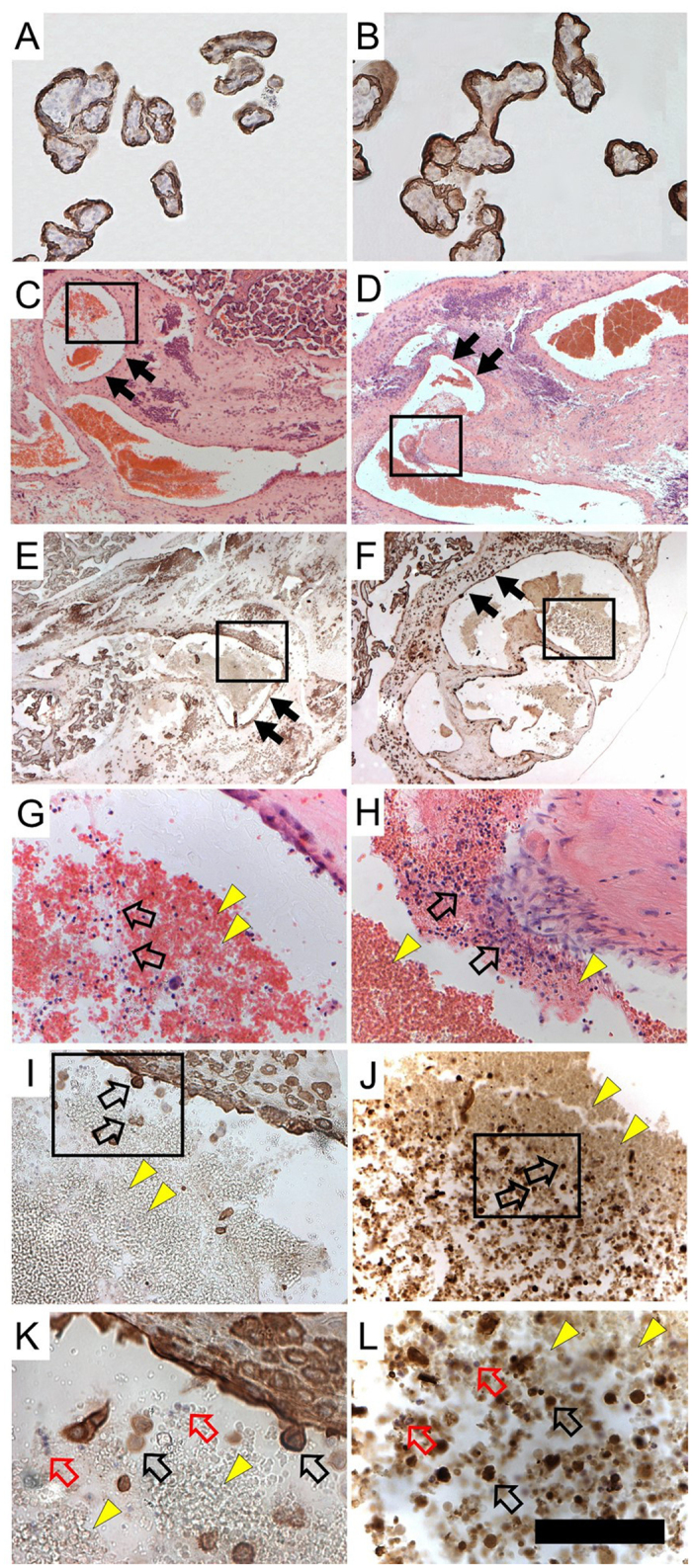
Photomicrographs of histological sections of a clinically normal placenta (**A,E,G,I,K**) and an IUGR placenta (**B,D,F,H,J,L**) showing villi and veins (embedded in intercotyledonary septa) in a plane parallel to the chorionic plate (**A,B**). Villous sections of a clinically normal (**A**) and an IUGR (**B**) placenta demonstrate specific labeling of trophoblast by cytokeratin 7 (CK7). (**C**–**F**) Tissue sections of veins (the vein walls are marked with black arrows) of a clinically normal (**C**,**E**) and an IUGR (**D**,**F**) placenta in low power overview. Sections shown in (**C**,**D**) were stained with hematoxylin-eosin (**H**,**E**), and sections shown in (**E**,**F**) were processed with immunohistochemistry using an anti-CK7 primary antibody. The black squares in (**C**–**F**) mark the regions shown at higher magnification in (**G**–**J**). The square in (**C**) corresponds to (**G**) the one in (**D**) corresponds to (**H**) the one in (**E**) corresponds to (**I**) and the square in (**F**) corresponds to (**J**). (**G**–**J**) Cells inside the veins of a clinically normal (**G**,**I**) and an IUGR (**H**,**J**) placenta. (**G**,**H**) Cells inside the veins stained with HE and (**I**,**J**) CK7 positive cells are shown. Yellow arrow heads mark erythrocytes, and clear arrows mark isolated mononuclear cells. The black squares in (**I** and **J**) mark the regions shown at even higher magnification in (**K** and **L**). The square in I corresponds to (**K**) and the square in (**J**) corresponds to (**L**). (**K**,**L**) Cells inside the veins of a clinically normal (**K**) and an IUGR (**L**) placenta. Yellow arrowheads mark erythrocytes, clear red arrows mark isolated mononuclear cells and clear black arrows mark CK7 positive particles, putatively trophoblast shedding. The scale bar in (**L**) is 50 *μ*m in (**K**,**L**), 100 *μ*m in (**A,B,G**–**J**) and 800 *μ*m in (**C–F**).

The dynamic flow models indicated areas of elevated wall shear stress at villous surfaces in the proximal IVS. To explore potentially elevated trophoblast shedding, we embedded a clinically normal and an IUGR placenta in paraffin *in toto*, divided the entire placenta block into four quarters and used one of these quarter placentas each for post-hoc histological analysis. The quarter placenta blocks were serially sectioned from the basal plate in a plane parallel to the chorionic plate. The serial sections were alternately stained either with hematoxylin and eosin (HE) or by immunohistochemical detection of cytokeratin 7 (CK7) as a trophoblast marker molecule (Fig. 4). In these sections, we could identify potential villous damage in IUGR, which appeared as cytokeratin-positive particles (partially as mononuclear particles) in the IVS but in a pronounced way in veins of intercotyledonary septa that drain the IVS.

## Discussion

### General Aspects and Limitations of the Model

The novel dynamic flow model of the present study extends far beyond the reach of the currently most advanced uteroplacental blood flow calculations^12^. It confirms the appearance of velocity jets inside the undilated arterial opening, but it substantially extends on aspects such as blood flow velocity and velocity jets in the IVS, directional flow (vortices), pressure analysis and wall shear stress. None of these aspects were previously simulated or determined. The morphological stage covered the spiralized artery and a substantial part of the post-arterial IVS and thus an assembly with the most relevant morphological features of the uteroplacental inflow region at the microscopic scale^7^. However, there are still limitations and specific assumptions which have to be kept in mind when interpreting the data of the present study. The model does not include fetoplacental vascularization or any aspects of the interplay of uteroplacental and fetoplacental circulations. It concentrates on a single uteroplacental inflow region, and the interplay with neighboring inflow regions was not considered. Although the height of 2 mm of the proximal IVS above the spiral artery opening considered in the model is substantial at the microscopic scale, this is at best one quarter of the full distance between the chorionic and the basal plates. The boundaries inevitably resulting from the still limited model dimension can cause boundary effects. The zero pressure setting of the models at the upper model boundaries of the proximal IVS is, e.g., decreasing the absolute pressure values (but not the pressure relations between the simulations) in proximity of the boundary such that the absolute pressure levels in the proximal IVS might be substantially lower than those in the proximal IVS *in vivo*. The present study was undertaken as a pilot which provides quantitatively formulated hypotheses for later experimental validation under clinical or laboratory conditions. Since the models of the present study translate strict physical rules into a single placental setting, experimental validation has to focus on the variability due to various settings, but is beyond the scope of the present study.

Where reasonably possible, the basic assumptions of the present model, e.g., the dimensions and flow data of the uterine artery^12^, the number of uterine spiral arteries^8^,^12^,^18^ per placenta and parameters for blood and artery dilation, were kept consistent with previous reports^12^ to ensure and maintain the best possible backward comparability. The total flow data of our model were chosen at the lower limit of our flow calculations, though knowing that higher flow would mean higher cargo delivery to the placenta. Flow was kept intentionally low to set a frame of conservative flow assumptions in terms of the occurrence of turbulence. Keeping these considerations in mind, the present study outlines four areas in which the simulation of pathological conditions indicated differences from the clinically normal situation that need to be discussed (Figs 1 and 2): (i) formation of high-velocity jets at the arterial opening and their projection into the neighboring proximal IVS; (ii) higher wall shear stress and its possible consequences, particularly at surface areas of the proximal IVS where the high-velocity jets hit the villous tree; (iii) the formation of recirculation zones (vortices) in the entry region; and (iv) regions of the proximal IVS with elevated intervillous blood pressure.

### High-Velocity Jets Project on the Villous Surface

The velocity jets observed in the pathological models of the present study developed inside the undilated opening of the spiral artery. The present model did not require assumptions on this opening because the morphologic stage was constructed from an undilated opening found in an IUGR placenta. Nevertheless, this real diameter corresponds well with the data used by others^12^. Our simulations confirm the Hagen-Poiseuille-based calculations of ref. 12 but are able to extend the simulation into the proximal IVS. The hypothesis of ref. 12 that the intravascular velocity jets extend substantially into the IVS could be confirmed by our extended simulations. All these findings together support our idea that dilation of the final segments of spiral arteries in human placentas can be considered to be a way to avoid or minimize the occurrence of velocity jets. This could be functionally essential because high-velocity regions such as the observed jets are contradictory to slowly progressing flow patterns known from other diffusion-governed organs with high metabolic activity, e.g., the liver^19^,^20^,^21^. Our models also showed that the occurrence of velocity jets was restricted to the peak systolic phases of the maternal heart cycle. This result indicates that not only the morphology of the arterial opening but also preuterine circulatory conditions could influence the occurrence of velocity jets.

### Wall Shear Stress and Trophoblast Shedding

In addition, the fluid dynamic model of the present study revealed increased wall shear stress at the villous surface in the inflow regions of the placenta (Fig. 1D–F; Movie S1a,b), particularly in regions that were reached by high-velocity jets. High wall shear stress indicates that some regions of the villous tree could become critically stressed. Likely, this has consequences for the sensitive syncytial trophoblast surface, which covers the villous tree and enables gas and nutrient exchange during pregnancy. Particularly in the vicinity of the velocity jets in IUGR and to a minor extent in IUGR/PE (Fig. 2), high wall shear stress could damage this epithelium or at least challenge its cellular turnover^22^,^23^. Effects of wall shear stress on trophoblast could potentially be mediated by certain mechanotransduction pathways that were recently identified in trophoblast^23^.

Post-hoc histological examination of uterine veins in intercotyledonary septa revealed evidence of increased damage at the trophoblast surface (Fig. 4). Increased amounts of cytokeratin-positive particles, partially also with a mononuclear appearance, were found in the intercotyledonary veins of a placenta from a patient with IUGR. The mixture of anuclear and seemingly mononuclear particles is not atypical for debris of syncytiotrophoblast. Such phenomena (including the appearance of mononuclear cytokeratin-positive particles) are known from protocols for isolating primary trophoblasts^24^. Because larger particles such as those observed here would stick in lung capillaries and be degraded there, elevated levels of (free) fetal DNA in the maternal circulation would be a possible endpoint of trophoblast shedding. This has indeed been reported as being associated with IUGR and IUGR/PE^25^,^26^,^27^,^28^.

### Vortex Flow Patterns Could Compromise Efficiency

Corresponding to the pressure drop at the arterial opening and the velocity jet in the center axis of the undilated spiral artery openings, vortices appeared in the pathological models, in direct proximity of the arterial opening. Such vortices were not described by other models and principally cannot be discovered using the limiting assumptions of laminar flow only by the Hagen-Poiseuille equation. In a vortex, the blood is circulating rather than slowly progressing along a surface. According to the data of the model of the present study, approximately 13.7–17.6% of the blood entering the placental IVS during the systolic peak phase of the maternal heart cycle could shunt into a vortex. Specifically, vortices can capture a substantial part of the inflow volume into cycling “dead volumes”, which would reach the feto-maternal exchange zone at the villous surface with delay and could possibly contribute to reduced, e.g., oxygen transfer^6^,^22^,^29^.

### Blood Pressure in the IVS

The simulations of the present study provided evidence that areas with higher than clinically normal blood pressure might extend deep into the IVS *in vivo*. However, the absolute values observed in the models in the proximal IVS should be interpreted with caution. They were influenced by boundary effects and the dimensions of the model and likely underestimated the true pressures in the IVS *in vivo* substantially and systematically. The pressures in the model of the present study would come closer to the *in vivo* values if (i) the proximal IVS would have substantially larger dimensions, ideally composing the entire villous zone between the basal plate and the chorionic plate, and (ii) the venous pressure in the draining veins would replace the zero pressure setting.

Although the blood pressure in the IVS *in vivo* might be higher than in the corresponding models, the difference in the outcome between the simulations of clinically normal and pathological conditions indicated a profound effect of the arterial opening on the pressure in the IVS. While pressure dropped more steeply at the arterial opening in the pathological cases than in the clinically normal situation, the end pressure in the proximal IVS of the IUGR and IUGR/PE conditions was higher than in the proximal IVS of the clinically normal situation. Increased blood pressure in the IVS, as indicated for the first time in the models of the present study, is unexpected in current pathogenetic concepts and could have a substantial impact on villous topology^30^ and placental function. These circumstances can lead to blood retention in the IVS next to the opening of the uterine spiral arteries. Because blood is not compressible, a large blood “lake” arising next to the opening of the uterine spiral arteries could operate as a blood pressure cuff in the IVS or could lead to a “dead volume”, which corresponds to the vortex formation outlined above.

*In vivo*, zones with blood pressure elevated above normal in the IVS are potentially extending far deeper in the placental tissue than was revealed by the dimensions of the fluid dynamics model of the present study (Fig. 1A–C). Elevated blood pressure in the IVS could potentially be an additional and independent factor compromising placental perfusion. The capillaries in the peripheral parts of the villous tree are directly exposed to the intervillous pressure and are kept open for fetal perfusion only by their internal pressure provided by the fetal heart.

These capillaries have no determinant of their diameter other than the relation of their capillary blood pressure to the blood pressure in the IVS^31^. It can be expected that the differences between the blood pressure in the IVS and the blood pressure in the fetal capillaries are discrete. Elevated blood pressure in the IVS, as suggested by the models of the present study, could thus be able to reduce fetal capillary diameters and increase fetal vascular resistance with the full 4th degree exponential power of diameter changes in this context^32^. Pressure peaks or constant pressure above a critical value in the IVS would tend (i) to compress these capillaries, preferably during the low-pressure phases of the fetal heart cycle, (ii) to substantially compromise fetal blood flow, and (iii) could possibly be the cause of umbilical reverse Doppler flow in the third trimester of gestation in pregnancies affected by severe IUGR.

Clinical Doppler umbilical flow parameters such as end diastolic flow and pulsatility index are clinical signals of micro-circulatory resistance properties inside the fetal vascular bed of the villous tree and are important for managing apparent disease to determine an optimal time of delivery^33^. Absent or reversed end diastolic umbilical flow could be interpreted in this context as a condition in which, e.g., an elevated intervillous pressure compresses intravillous capillaries and contributes to critical reduction of fetal blood flow and could even be a factor helping to shift arterial volume back at the end of the fetal diastole^4^,^33^,^34^.

## Conclusion

The dynamic flow model implemented in the present study indicates that insufficient remodeling of uterine spiral arteries could have a multitude of effects on the blood flow in spiral arteries and the IVS. The villous surface appears to be exposed to increased mechanical stress by velocity jets and elevated wall shear stress, which correspond to intensified trophoblast shedding and elevated levels of fetal DNA in maternal blood. The present study underlines this concept by histopathologically demonstrated increased trophoblast shedding. Potentially even more important are those modifications of the blood flow in the IVS which have the potential to directly impair the placental exchange functions. These are (i) turbulent flow conditions, particularly vortices at the arterial openings, which are an equivalent of functional “dead volumes” with suboptimal exchange capacity, and (ii) elevated blood pressure in the IVS, which could potentially become a risk for fetoplacental perfusion as soon as fetal capillaries become compressed or even collapse. In view of the concept of ischemic placental disease, the present study demonstrates possible pathogenetic mechanisms that are capable of linking the early origins (insufficient arterial remodeling) to the considerably later development of clinically symptomatic placental disease^1^,^35^,^36^.

## Materials and Methods

### Tissue sources and ethical approval

All clinical and morphological examinations and tissue collections were part of a larger study approved by the ethics committee of the Ludwig-Maximilians-University of Munich (LMU Munich, Germany) under number 478–12. The placentas were collected at the Department of Obstetrics and Gynecology of the hospital Dritter Orden, Munich, Germany. The placentas were allocated to clinical groups by the obstetricians based on clinical information regarding the pregnancy and delivery. The collection included clinically normal placentas and placentas of pregnancies with intrauterine growth retardation (IUGR) with absence or presence of preeclampsia (PE). An IUGR was diagnosed if the growth data of the fetus determined by ultrasound (e.g., body length and head circumference) were above the 10th growth percentile during the first two trimesters and then dropped below the 10th growth percentile^4^,^11^. In addition to IUGR, PE was defined by arterial hypertension (≥140/90 mmHg) with onset beyond the 20th week of gestation or substantial proteinuria (≥300 mg/24 h) after the 20^th^ week of gestation^4^,^11^. The study protocol allowed for the inclusion of clinical data such as Doppler data if anonymity is maintained. Placentas were collected after informed consent of mothers/parents was obtained. Placentas were excluded when no informed consent of the mothers/parents could be obtained, when the language skills of the mothers/parents limited understanding of information regarding the study, or when psychiatric problems or any other condition caused doubts regarding the ability of the mothers/parents to independently decide. All work was conducted according to relevant guidelines and regulations, and all data were anonymized. Additional details are outlined in Supplementary Information (*Sources of tissue and clinical data; ethical approval*).

### Tissue processing for morphology and Doppler data

During routine histopathological examination, a long segment of a spiral artery and the adjacent region of the villous tree and intervillous space were located in a placenta of a patient with IUGR (Table S1). Serial sectioning and reconstruction were used to three-dimensionally reconstruct the spiral artery and the inflow region proximal to the arterial opening (proximal IVS). Technical details of this procedure are outlined in Supplementary Information (*Generation of the 3D tissue reconstruction*).

For post-hoc histological analysis of potential tissue damage in specific areas of the villous tree, one additional placenta from a clinically normal pregnancy and one additional placenta of a patient with IUGR (Table S1) were used. Both placentas were fixed and embedded in paraffin, each *in toto*. Serial sections, of one quarter of each placenta, were taken in a plane that was parallel to the chorionic plate, beginning at the basal plate and stained alternately with hematoxylin-eosin or used for immunohistochemical detection of the trophoblast marker molecule cytokeratin 7^7^. Technical details of this procedure are outlined in Supplementary Information (*Tissue processing for post-hoc histological analysis; Immunohistochemistry protocol*). The Doppler data of uterine arteries used in the dynamic flow model of the present study were collected from three patients with either uncomplicated pregnancy, IUGR or IUGR/PE, respectively (Doppler data and clinical data of these patients are shown in Fig. 3).

### Calculated blood flow based on velocity waveforms from the uterine artery

Blood flow in the left (l) and right (r) uterine artery 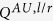 was calculated using the velocity 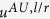 coming from Doppler ultrasound measurements in the left and right uterine artery and a cross sectional area *A*^*AU*^ with a diameter of d*AU* = 3.0 mm^18^,^37^ as


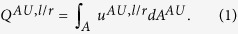


We used three typical waveforms from clinically normal, IUGR and IUGR/PE pregnancies (collected by Tanja Ruebelmann, Dritter Orden hospital). Assuming that this flow is distributed equally to n = 130 uterine spiral arteries in the placenta^38^, blood flow through a single uterine spiral artery reads as


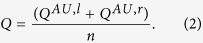


With a mean inlet diameter 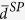 = 0.34 mm of the three-dimensionally reconstructed uterine spiral artery, this results in velocity waveforms at the uterine spiral artery inlet of


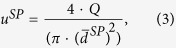


which was applied at the artery inlet in the simulations.

### Flow simulation through variable geometries of uterine spiral arteries

Blood flow in the fluid domain was governed by the incompressible Navier-Stokes equations


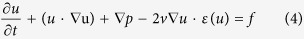



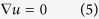


where the velocity is denoted as **u**, the kinematic pressure as *p*, and the kinematic viscosity as *ν*. The vector **f** represents a given body force, and


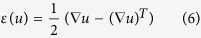


represents the deformation rate tensor of the fluid. These equations are solved via stabilized finite elements^39^,^40^ in our in-house code, which has been used successfully in several biomedical flow applications^41^,^42^.

In this study, blood was modeled as a Newtonian fluid with a kinematic viscosity of 6 mPas, which corresponds to the upper viscous limit in^12^ and a density of *ρ* = 1.055*·*10*−*6 g/mm^3^. At the artery inlet, velocity waveforms extracted from Doppler ultrasound measurements at the left AU were prescribed (see previous section). At the walls of the artery and the bottom of the placenta, no-slip boundary conditions were used, and at the sides of the intervillous space, slip boundary conditions were used. At the top surface of the modeled IVS, a zero-traction boundary condition was applied. All computations were performed with a time step of ∆*t* = 10^*−*4 ^*s* and a convergence criterion of 10^*−*5^ in the nonlinear residual. The simulations were performed on the SuperMuc Petascale System (Project “pr83te: High Performance Methods for Computational Fluid Dynamics including Multiphysics Scenarios”) of the Leibniz Supercomputing Center (Garching, Germany) and were analyzed and visualized using Paraview software^43^,^44^ in the morphological stage of the present study, which is in agreemement with general anatomic data^45^,^46^,^47^,^48^,^49^,^50^ (for details see Supplementary Information).

## Additional Information

**How to cite this article:** Roth, C. J. *et al*. Dynamic modeling of uteroplacental blood flow in IUGR indicates vortices and elevated pressure in the intervillous space – a pilot study. *Sci. Rep.* **7**, 40771; doi: 10.1038/srep40771 (2017).

**Publisher's note:** Springer Nature remains neutral with regard to jurisdictional claims in published maps and institutional affiliations.

## Supplementary Material

Supplementary Information

Supplementary Movie S1a

Supplementary Movie S1b

Supplementary Movie S2

Supplementary Movie S3a

Supplementary Movie S3b

## Figures and Tables

**Table 1:** Table 1 shows the clinical and macroscopic data of the placentas of the pregnancies from which the Doppler data shown in Fig. 3 were obtained (GA, gestational age; BW, birth weight; PW, placental weight; PW/BW, placental birth weight ratio; LD, longest diameter of the placenta; SD, shortest diameter of the placenta; surface area of the placenta; roundness of the placenta; and thickness of the placenta).

	Clinically normal	IUGR	IUGR/PE
GA [weeks]	38.5	33.6	33.3
BW [g]	3130	1620	1470
PW [g]	488	277	339
PW/BW ratio	0.16	0.17	0.23
LD [cm]	17.5	15.0	19.5
SD [cm]	15.0	13.0	10.5
surface area [cm^2^]	825	613	643
roundness	1.17	1.15	1.86
thickness [cm]	2.10	2.00	1.30
