# Supplementary material for: Dynamic modeling of uteroplacental blood flow in IUGR indicates vortices and elevated pressure in the intervillous space – a pilot study
Source: Sci Rep. 2017 Jan 19;7:40771. doi: 10.1038/srep40771 (PMC5244422; doi:10.1038/srep40771)
Supplement: Supplementary Information [file srep40771-s1.pdf]

Dynamic modeling of uteroplacental blood flow in IUGR indicates vortices and elevated pressure in the intervillous space – a pilot study

Christian J. Roth, Eva Haeussner, Tanja Ruebelmann, Franz von Koch, Christoph Schmitz, Hans-Georg Frank and Wolfgang A. Wall

Supplementary Information

Materials and Methods

Sources of tissue and clinical data; ethical approval. Clinical and macroscopic data were determined using standard protocols [45]. The dynamic flow model of the present study used a morphological stage that included a uterine spiral artery and adjacent villous trees (i.e., the inflow region) of a placental cotyledon of a placenta of a patient with IUGR (**Table S2**). This uterine spiral artery was found during routine histopathological examination.

For post-hoc histological analysis of trophoblast shedding, one additional placenta from a clinically normal pregnancy and one additional placenta of a patient with IUGR were used (**Table S2**). These placentas were embedded and processed for histology *in toto* , i.e. without sampling of probes in a specific non-routine manner (for details, see the section on tissue processing below).

The Doppler data of uterine arteries used in the dynamic flow model of the present study were collected from three representative patients with either uncomplicated pregnancy, IUGR, or IUGR with the presence of preeclampsia (IUGR/PE), respectively; **Figure 3** in the main text).

Generation of the 3D tissue reconstruction. Tissue samples for routine histology were fixed in phosphate-buffered formalin (4.5%) for at least 24 h. The samples were processed routinely and embedded in paraffin. Sections were stained with hematoxylin/eosin (HE) [45]. In one section of a tissue sample of a placenta of a pregnancy with IUGR, a uterine spiral artery still attached to the basal plate was found (**Figure S1A**). This tissue block was serially sectioned in 10 µm thick tissue sections (a total of 120 sections). Every fifth section out of the 120 sections was automatically scanned using 10x objective. Automated scanning was based on a M2 Axio Imager (Zeiss, Goettingen, Germany) microscope equipped with a two-axis computer-controlled stepping motor

system (4x3 XY), automated slide loader with proScan III stage (Prior Scientific, Jena, Germany) and color digital camera (2/3 CCD chip 1,4 MP, 1388x1049 pixel; MBF Bioscience, Williston, VT, USA), and focus encoder (MBF Bioscience). The entire system was controlled by the software Stereo Investigator (MBF Bioscience). Then, the scan files were aligned with Amira software (version 5.5.0; Visualization Sciences Group, FEI Company, Burlington, MA, USA). All images (size: 2560x1518 pixels; resolution: 5.9 µm/pixel, section thickness: 10 µm; distance between sections: 50 µm) were converted to DICOM and stacked to generate a three-dimensional data set (**Figure S1B**). From these data, blood was segmented in the region of the uterine spiral artery (marked red in **Figure S1B**), and the intervillous space (IVS) at the opening of the artery was marked yellow in **Figure S1B** using Mimics software (version 16.0; Materialise, Leuven, Belgium). The segmented geometry was closed using symmetry conditions to account for a small part of the artery that was not contained in the placenta sample (using Mimics software). Additionally, a second remodeled artery geometry was created by dilating the outlet radius by a factor of 3 within a smooth transition zone in the last 3 mm of the artery length according to [12] (using Mimics software). The resulting geometry of both arteries was connected to the IVS, improved in quality [46] and exported in STL file format. The final arterial length was approximately 10 mm with a mean inlet diameter of 0.34 mm and an outlet diameter of 1.15 mm for the clinically normal remodeled uterine spiral artery, and 0.39 mm for the pathological artery, which corresponds well to previous observations [12, 47]. The IVS proximal to the artery opening and included in the stage (proximal IVS) had dimensions of 3.0x2.5x2.0 mm and was approximately the size that is supplied with blood via a single uterine spiral artery [12]. A three-dimensional tetrahedral mesh was generated using Gmsh (version 2.9.3; freeware by [48]) and consisted of 1,548,144 tetrahedral elements in the clinically normal case and 1,502,567 elements in the pathological cases.

Tissue processing for post-hoc histological analysis. For post-hoc microscopic analysis of trophoblast shedding, placentas of a clinically normal pregnancy and an IUGR pregnancy were used (**Table S1**). These placentas were processed for histology in an innovative, non-routine manner. To this end the placentas were fixed in 4.5% phosphate-buffered formaldehyde (Roti-Histofix, Carl Roth, Karlsruhe, Germany) for 7 days and embedded in paraffin (Paraplast, melting point 56−58°C; Leica No. 39602012; Wetzlar, Germany), each placenta without tissue sampling as a whole. After fixation, embedding began with passage through a serial stepwise ethanol gradient

from 50% ethanol for 6 h, 70% ethanol for 6 h, second time 70% ethanol for 12 h, 80% ethanol for 12 h, 96% ethanol for 12 h, and two times 100% ethanol for 15 h. From pure ethanol, the placentas were transferred in xylene (two times in xylene for 12 h and 8.5 h). From xylene, the placentas were transferred to paraffin at 65°C via three infiltration steps for 24 h each. A special form with a diameter of 32 cm was used for the entire embedding procedure. To enable sectioning, the paraffin block containing the whole placenta was cut into four quarters. Each quarter block was oriented for sectioning such that sections were taken in a plane parallel to the chorionic surface, starting at the basal plate of the placenta (Microtom, Polycut S; Reichert Jung, Wetzlar, Germany). From the level of the first appearance of tissue in the section onwards, serial sections with a thickness of 20 µm were prepared and collected between numbered sheets of paper. All sections were mounted on oversize object slides (120x150x1 mm). For standard hematoxylin-eosin (HE), the object slides were coated with 0.5% alum chrome gelatin (7.5 g gelatin [Prolabo VWR, article No. 24360.233; Darmstadt, Germany]; 0.75 g chromium (III) potassium sulfate dodecahydrate [Merck, article No. 1.01036.0250; Darmstadt, Germany]).

For immunohistochemistry, the object slides were coated with poly-L-lysine (Sigma, article No. P8920, Munich, Germany). For coating with poly L-lysine, the object slides were degreased in acetone for 10 min, incubated with poly-L-lysine (1:10 in distilled water) for 30 min and dried at 60°C for 1 h. Prior to tissue staining or immunohistochemical processing, the mounted paraffin sections were deparaffinized by transferring from xylene (two times for 10 min and 5 min in xylene) through a descending ethanol series (100% ethanol two times for 3 min, 96% ethanol for 3 min, 80% ethanol for 3 min, 70% ethanol for 3 min, 50% ethanol for 3 min, and finally distilled water for 3 min). Every 10th section was stained with standard hematoxylin-eosin (HE) and the following serial section by immunohistochemistry (see below). Trophoblast nature of cellular elements in the sections was ensured by using anti-cytokeratin 7 (CK7, Dako Clone OV TL12/30 Code M7018, monoclonal mouse; Santa Clara, CA, USA) reactivity.

Immunohistochemistry protocol. The sections were washed in PBS for 5 min and incubated with 0.1% protease (Sigma, article No. P4630; 0.01 g of protease in 10 ml of TBS buffer [AppliChem, article No. A1086; Darmstadt, Germany]) for 20 min at 37°C. Then, the sections were rinsed briefly in tap water and incubated for 5 min in PBS. To block endogenous peroxidase, the sections were incubated in 0.3% H_2O_2 for 30 min (Merck, article No. 1.07209) and washed in PBS for 5 min. For the next steps, we used the ZytoChem-Plus HRP kit (Zytomed Systems, article No. HRP125;

Berlin, Germany) according to the manufacturer's protocols. The first antibody (1:50 CK7 in PBS buffer) was incubated for 1 h at 37°C and then washed three times for 5 min in PBS. Finally, the antibody was visualized by DAB (Sigma, article No. D5905) for visualization of peroxidase (10 min). All stained sections were washed briefly in tap water and two times for 5 min in distilled water. Next, counterstaining with hematoxylin was achieved by short incubation in the staining solution (30 sec) and bluing in tap water for 5 min. Sections were passed through an increasing alcohol series in xylene and mounted in DPX (Prolab VWR, article No. 360294H) under oversize cover slips.

All tissue sections were examined using an Axiophot microscope (Axiophot with AxioCam HRc, Zeiss) with a 2.5x objective for an overview and 20x and 40x objectives for detailed views. The stained sections were sequentially examined. Venous drains were mostly available in the intercotyledonary septa and exhibited trophoblast remodeling in the media (**Figure 4** in the main text) [**7, 49, 50**]. The volume of such intercotyledonary venous vessels was visually examined for possible cytokeratin-positive trophoblast deportations.

Input parameters for blood flow. All governing parameters for blood flow were derived from Doppler measurements at the uterine artery of the three pregnancies (normal, IUGR, and IUGR/PE) listed in **Figure 3** in the main text. Doppler measurements originally provided waveforms for blood flow velocity $u^{AU,l/r}$ in (cm/s) in the arteria uterina (*AU*) over three maternal heart cycles (**Figure 3** in the main text, left y-axis). The superscripts *l/r* indicate the left and right uterine arteries, respectively. Considering the uterine artery as a vessel with a circular cross section area A^{AU} with a diameter of d^{AU} = 3.0 mm [**18, 37**], blood flow in the left and right arteria uterina was computed as

$$Q^{AU,l/r} = \int_A u^{AU,l/r} dA^{AU} .$$

The computed total blood volume provided to the placenta is the sum of the flows coming from the left and right uterine artery and was determined using the above equation to be in the range of 339 to 593 ml/min along the three ultrasound waveforms (clinically normal, IUGR, and IUGR/PE). These values are in good agreement with values reported in [**18, 37**].

Assuming that this total blood flow provided to the placenta distributes equally to a number of n=130 spiral arteries, which is the average of the values provided in the literature (n=60 in [12] and n=200 in [18]), the blood flow through a single uterine spiral artery reads as

$$Q = \frac{(Q^{AU,l} + Q^{AU,r})}{n}$$

and was used to feed the simulations of blood flow in this study (**Figure 3** in the main text). Compared to [12], our values for maximum blood flow *Q* through a single spiral artery (**Table S2**) are conservative due to the larger number of spiral arteries assumed in our models [12].

The full time span of the three visualized waveforms (normal, IUGR, and IUGR/PE) during the three maternal heart cycles was simulated in each case, which led to the following simulation setups: (i) *clinically normal situation:* dilated spiral artery geometry with clinically normal waveform; (ii) *IUGR:* un-dilated spiral artery geometry with IUGR waveform; and (iii) *IURG/PE:* un-dilated spiral artery geometry with IUGR/PE waveform. During the first maternal heart cycle, flow was allowed to fully develop from the initial resting state, and the second maternal heart cycle provided representative rheological properties in the spiral artery. Therefore, the estimates of the models were all taken from the values of the 2nd maternal heart cycle, i.e., the interval of 1.2 to 2.0 s. This interval was also the basis for the figures and movies and is indicated by the black bar in **Figure 3** in the main text**.**

Tables

Table S1. Macroscopic clinical data of the placentas that were processed for histology. This includes the tissue sample that provided the morphological stage of the present study (three-dimensional [3D] reconstruction of a spiral artery and the corresponding proximal IVS of an IUGR placenta [see **Fig.S2**]) and the two placentas that were non-routinely processed post-hoc to analyze trophoblast shedding (GA, gestational age; BW, birth weight; PW, placental weight; PW/BW, placental birth weight ratio; LD, longest diameter of the placenta; SD, shortest diameter of the placenta; surface area of the placenta; roundness of the placenta; thickness of the placenta).

	**3D reconstruction of an IUGR placenta with attached spiral artery**	**Post-hoc histological analysis**	
		**clinically normal**	**IUGR**
GA [weeks]	27.5	39.0	37.6
BW [g]	745	4450	2225
PW [g]	235	684	331
PW/BW ratio	0.32	0.15	0.15
LD [cm]	15.0	22.5	17.0
SD [cm]	13.5	22.0	12.0
surface area [cm^2]	636	1555	641
roundness	1.11	1.02	1.42
thickness [cm]	1.50	1.60	1.50

Table S2. Input and output parameters of the dynamic flow model implemented in the present study. The parameters correspond to the three clinical conditions (clinically normal, intrauterine growth restriction (IUGR), and IUGR/preeclampsia (IUGR/PE)).

**Dynamic Flow Model**	**clinically normal**	**IUGR**	**IUGR/PE**
Number of spiral arteries [38]	130	130	130
Spiral arterial inlet diameter [mm]	0.34	0.34	0.34
Mean flow per heart cycle (Q) [ml/s]	0.031	0.027	0.017
Max. flow per heart cycle (Q) [ml/s]	0.045	0.047	0.036
Max. pressure at artery inlet [mm Hg]	80	123	107
Pressure drop in arterial opening [mm Hg]	0.48	9.72	6.95

Figures

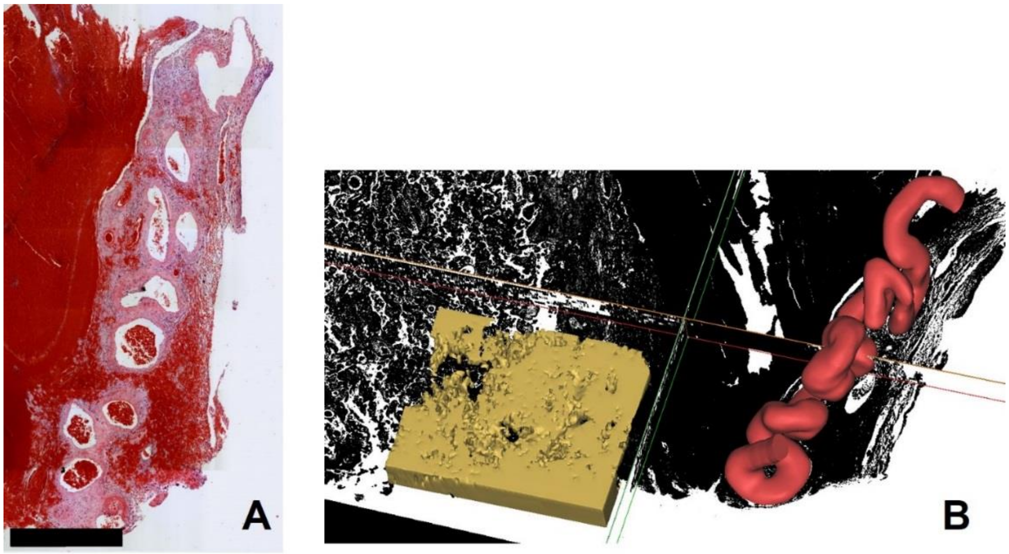

Figure S1. (A) Photomicrograph of the spiral artery used in the present study for preparation of the morphological stage, stained with hematoxylin-eosin (HE). The scale bar represents 1200 μm. (B) Reconstructed spiral artery (red) and proximal intervillous space (yellow) based on histological images.

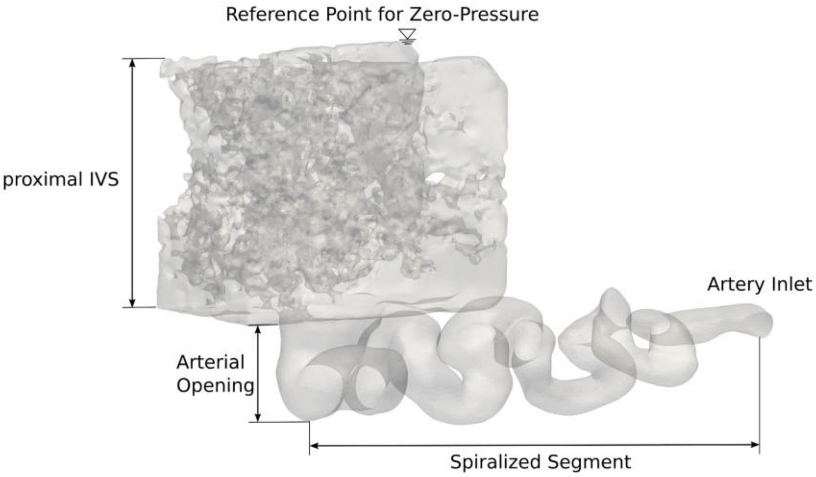

Figure S2. Overview on the various parts of the morphological stage of the dynamic flow model of the present study. The stage is kept in light gray and was prepared by three-dimensional reconstruction and subsequent modification. It contains the uterine spiral artery with three domains (artery inlet, spiralized segment and arterial opening) and the intervillous space proximal to the arterial opening (proximal IVS). At the upper boundary of the proximal IVS, the blood pressure inside the IVS was by definition set to zero at the reference point.

Movies

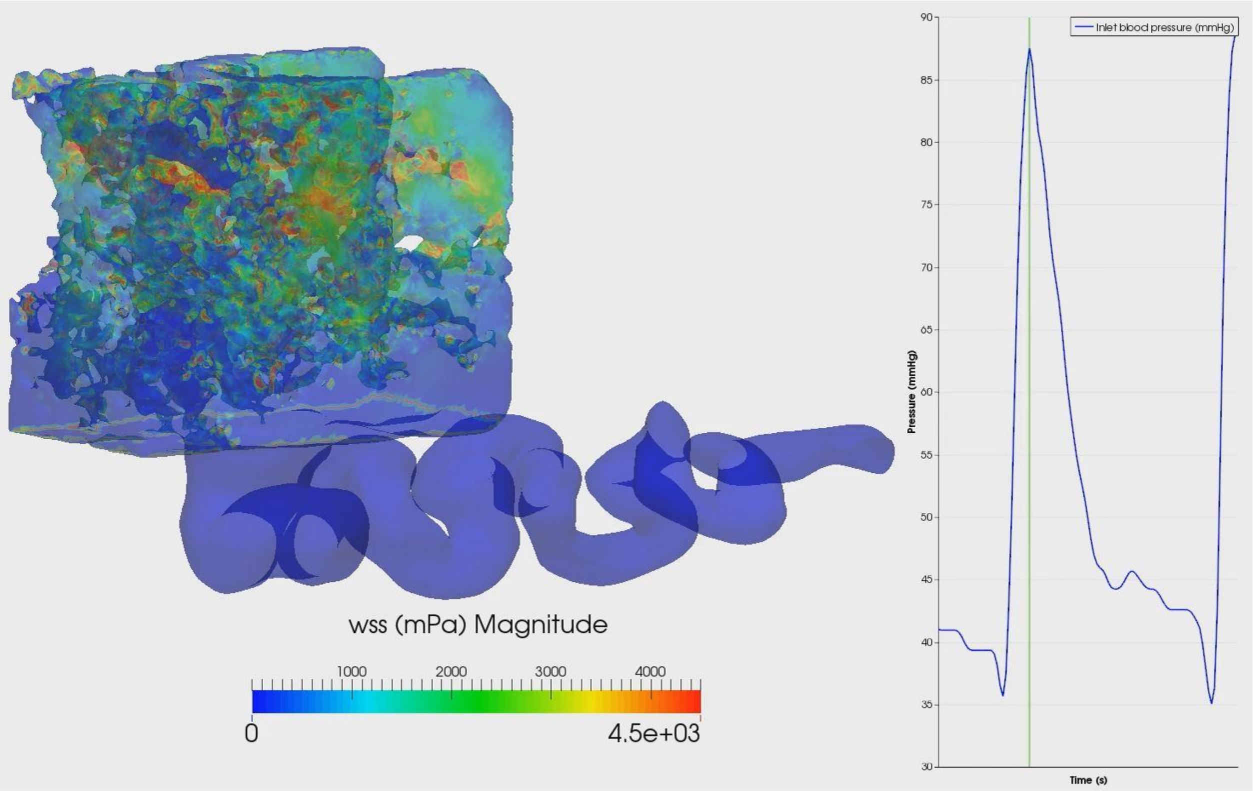

Still Image of Movie S1a. The movie shows the entire morphological stage of the model under clinically normal conditions with wall shear stress (in mPa, color coded, see scale inside the movie), including the spiralized uterine artery in the lower part and the cuboid block of the proximal IVS in the upper part. The graph at the right side of the movie shows the blood pressure at the arterial inlet during one maternal heart cycle. A green line is progressing with the maternal heart cycle in this graph and indicates the actual point in time of the dynamic flow model.

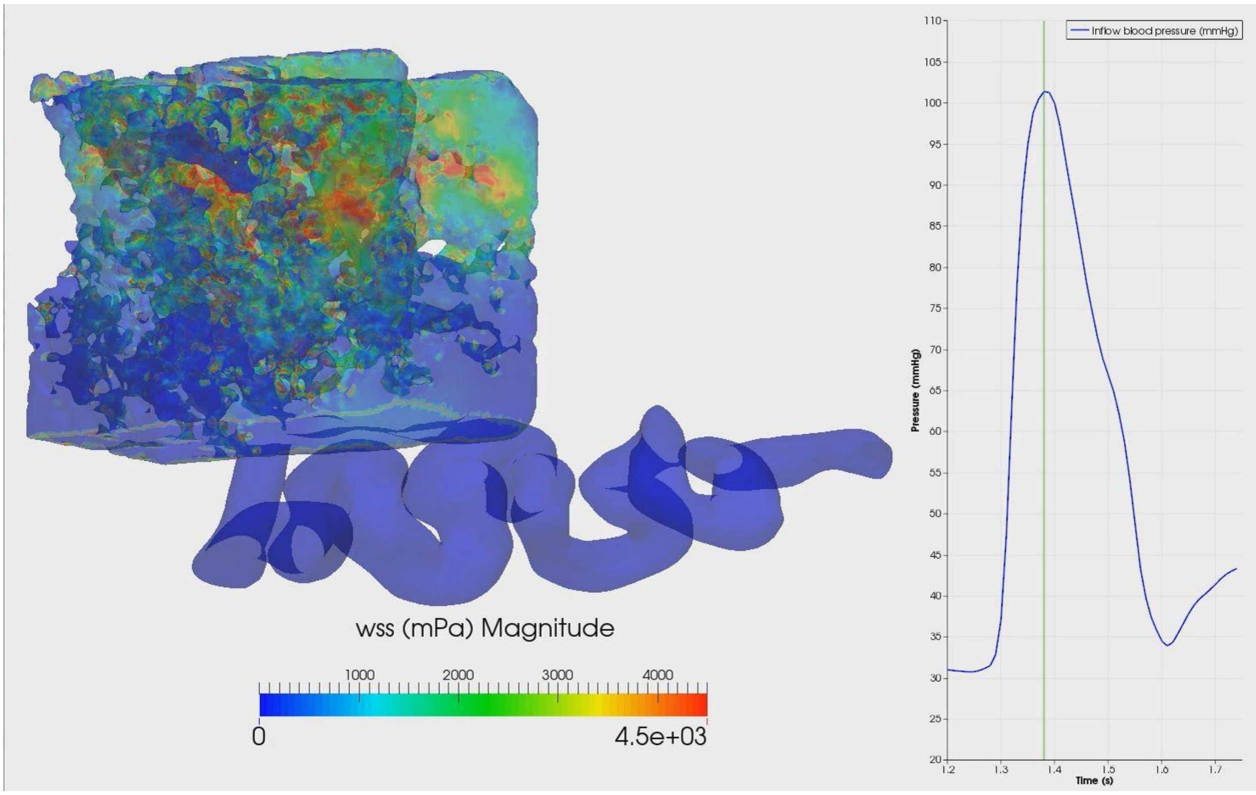

Still Image of Movie S1b. The movie shows the entire morphological stage of the model under intrauterine growth retardation conditions with wall shear stress (in mPa, color coded, see scale inside the movie), including the spiralized uterine artery in the lower part and the cuboid block of the proximal IVS in the upper part. The graph at the right side of the movie shows the blood pressure at the arterial inlet during one maternal heart cycle. A green line is progressing with the maternal heart cycle in this graph and indicates the actual point in time of the dynamic flow model.

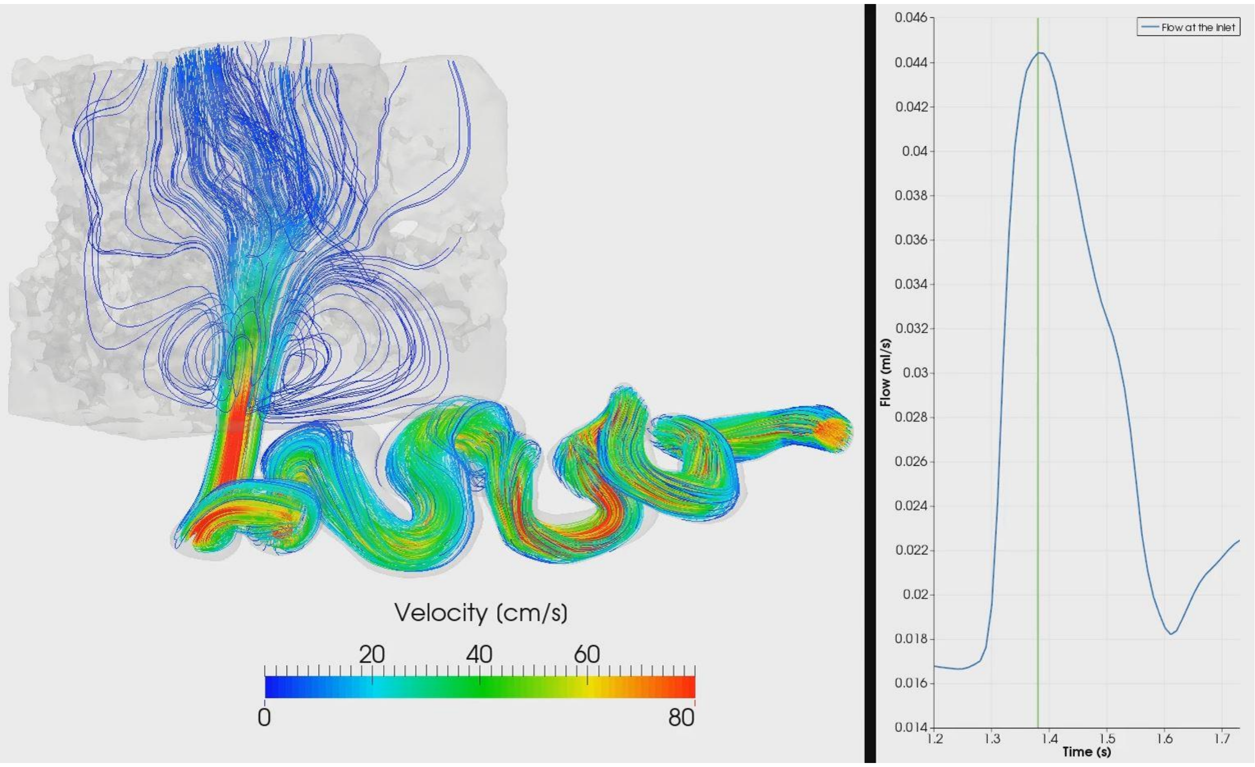

Still Image of Movie S2. The movie shows the entire morphological stage of the model under intrauterine growth retardation conditions, including the spiralized uterine artery in the lower part and the cuboid block of the proximal IVS in the upper part. Velocity streamlines are shown (in cm/s, velocity is color coded, see scale inside the movie); each streamline corresponds to the course of one single erythrocyte. The graph at the right side of the movie shows the blood flow at the arterial inlet during one maternal heart cycle. A green line is progressing with the maternal heart cycle in this graph and indicates the actual point in time of the dynamic flow model. Clear formation of the vortex in the proximal IVS starts once the critical flow value of 0.035 ml/s is exceeded at the artery inlet.

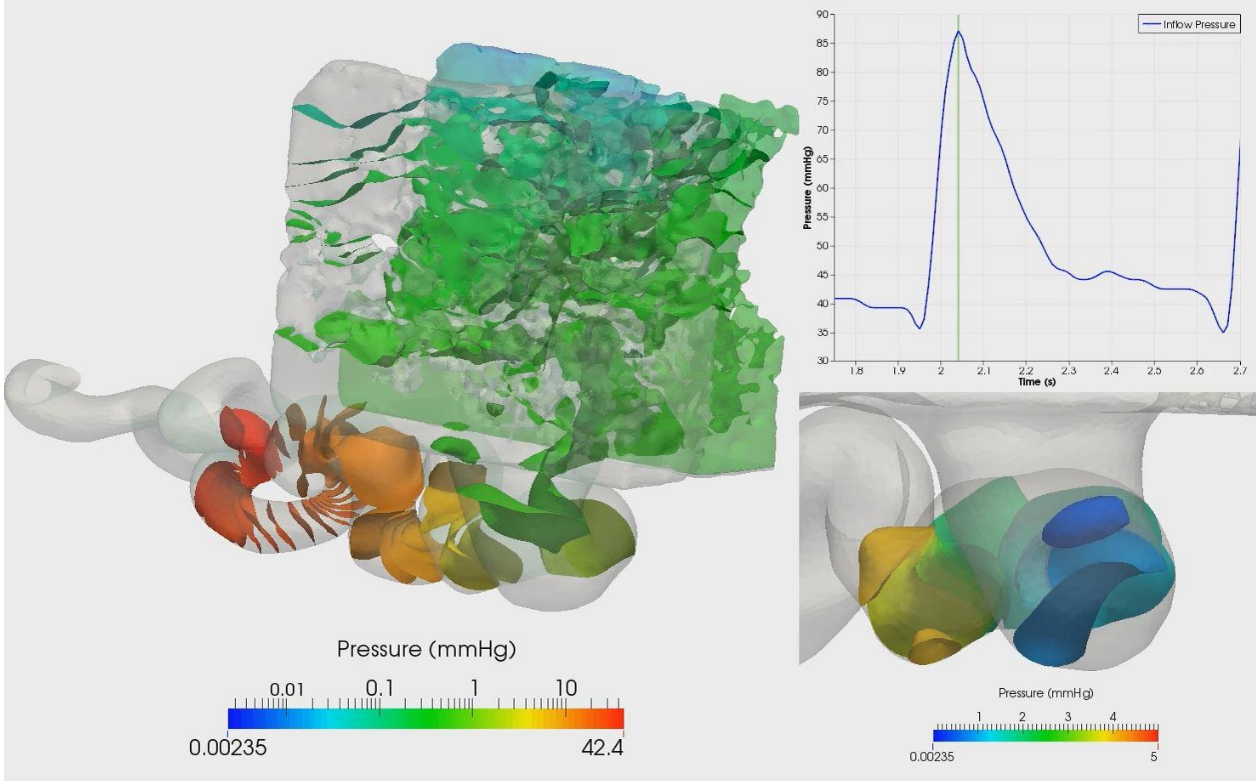

Still Image of Movie S3a. The movie shows the entire morphological stage of the model under clinically normal conditions, including the spiralized uterine artery in the lower part and the cuboid block of the proximal IVS in the upper part. Iso-pressure surfaces are shown (in mmHG, pressure is color coded, see scale inside the movie). Iso-pressure surfaces connect voxels with the same pressure value. The graph at the upper right side of the movie shows the blood pressure at the arterial opening during one maternal heart cycle. A green line is progressing with the maternal heart cycle in this graph and indicates the actual point in time of the dynamic flow model. The graph at the lower right side of the movie shows the blood pressure at the arterial opening in higher magnification with a specific color scale (below the movie at the lower right side).

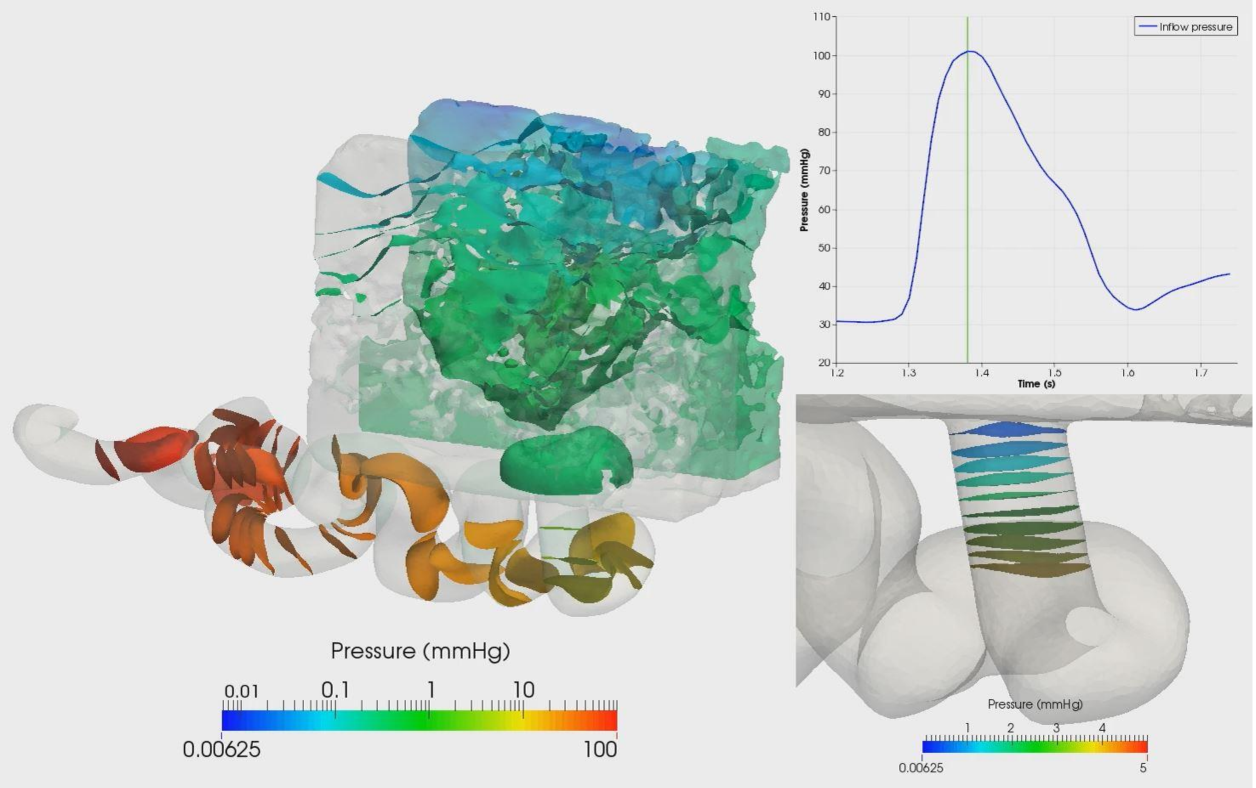

Still Image of Movie S3b The movie shows the entire morphological stage of the model under intrauterine growth retardation conditions, including the spiralized uterine artery in the lower part and the cuboid block of the proximal IVS in the upper part. Iso-pressure surfaces are shown (in mmHG, pressure is color coded, see scale inside the movie). Iso-pressure surfaces connect voxels with the same pressure value. The graph at the upper right side of the movie shows the blood pressure at the arterial opening during one maternal heart cycle. A green line is progressing with the maternal heart cycle in this graph and indicates the actual point in time of the dynamic flow model. The graph at the lower right side of the movie shows the blood pressure at the arterial opening in higher magnification with a specific color scale (below the movie at the lower right side).

References

1. Ananth, C.V. Ischemic placental disease: A unifying concept for preeclampsia, intrauterine growth restriction, and placental abruption. Ischemic placental disease. Semin Perinatol. 38:131-132 (2014).

2. Ananth, C.V. & Friedman, A.M. Ischemic placental disease and risks of perinatal mortality and morbidity and neurodevelopmental outcomes. Semin Perinatol. 38:151- 158 (2014).

3. Parker, S.E. & Werler, M.M. Epidemiology of ischemic placental disease: A focus on preterm gestations. Ischemic placental disease. Semin Perinatol. 38:133-138 (2014).

4. Rath, W. Geburtshilfe und Perinatologie. 2nd ed. (Thieme, Stuttgart, 2010).

5. Chaddha, V., Viero, S., Huppertz, B. & Kingdom, J. Developmental biology of the placenta and the origin of placental insufficiency. Semin Fetal Neonatal Med. 9:57-69 (2004).

6. Roberts, J.M. Pathophysiology of ischemic placental disease. Semin Perinatol. 38:139-145 (2014).

7. Benirschke, K., Burton, G.J. & Baergen, R.N. Pathology of the human placenta. 6th ed. (Springer, Berlin and London, 2012).

8. Pijnenborg, R., Brosens, I. & Romero, R. Placental bed vascular disorders. Basic science and its translation to obstetrics. (Cambridge University Press, Cambridge, 2010).

9. Bakker, P. & van Geijn, H.P. Uterine activity: implications for the condition of the fetus. J Perinat Med. 36:30-37 (2008).

10. Pijnenborg, R., Vercruysse, L. & Hanssens, M. Fetal-maternal conflict, trophoblast invasion, preeclampsia, and the red queen. Hypertens Pregnancy. 27:183-196 (2008).

11. Polin, R.A. & Fox, W.W. Fetal and neonatal physiology. 4th ed. (Saunders, Philadelphia, 2011).

12. Burton, G.J., Woods, A.W., Jauniaux, E. & Kingdom, J.C.P. Rheological and Physiological Consequences of Conversion of the Maternal Spiral Arteries for Blood Flow during Human Pregnancy. Placenta 30:473-482 (2009).

13. Wooding, P. & Burton, G.J. Comparative Placentation. (Springer, Berlin and Heidelberg, 2008).

14. Pennati, G., Socci, L., Rigano, S., Boito, S. & Ferrazzi, E. Computational Patient-Specific Models Based on 3-D Ultrasound Data to Quantify Uterine Arterial Flow During Pregnancy. IEEE Transactions on Medical Imaging 27: 1715–1722 (2008).

15. Ferrazzi, E., Rigano, S., Padoan, A., Boito, S., Pennati, G. & Galan, H.L. Uterine Artery Blood Flow Volume in Pregnant Women with an Abnormal Pulsatility Index of the Uterine Arteries Delivering Normal or Intrauterine Growth Restricted Newborns. Placenta 32: 487–492 (2011).

16. Rigano, S., Ferrazzi, E., Boito, S., Pennati, G., Padoan, A. & Galan, H. Blood Flow Volume of Uterine Arteries in Human Pregnancies Determined Using 3D and Bi-Dimensional Imaging, Angio-Doppler, and Fluid-Dynamic Modeling. Placenta 31: 37–43 (2010).

17. Konje, J.C., Kaufmann, P., Bell, S.C. & Taylor, D.J. A longitudinal study of quantitative uterine blood flow with the use of color power angiography in appropriate for gestational age pregnancies. Am J Obstet Gynecol 185:608–613 (2011).

18. Burbank, F. Fibroids, Menstruation, Childbirth, and Evolution: The Fascinating story of uterine blood vessels. (Wheatmark, Tucson, 2009).

19. Munk, O.L., Bass, L., Feng, H. & Keiding, S. Determination of regional flow by use of intravascular PET tracers: microvascular theory and experimental validation for pig livers. J Nucl Med. 44:1862-1870 (2003).

20. Seifalian, M., Stansby, G.P., Hobbs, K.E.F., Hawkes, D.J. & Colchester, A.C.F. Measurement of Liver Blood Flow: A Review. HPB Surg. 4:171-186 (1991).

21. Rappaport, A.M. Hepatic blood flow: morphologic aspects and physiologic regulation. Int Rev Physiol. 21:1-63 (1980).

22. Clark, A.R., Lin, M., Tawhai, M., Saghian, R. & James, J.L. Multiscale modelling of the feto-placental vasculature. Interface focus. 5:20140078 (2015).

23. Miura, S., Sato, K., Kato-Negishi, M., Teshima, T. & Takeuchi, S. Fluid shear triggers microvilli formation via mechanosensitive activation of TRPV6. Nat Commun. 6:8871 (2015).

24. Frank, H.G., et al. Cell culture models of human trophoblast–primary culture of trophoblast–a workshop report. Placenta 21 Suppl. A:S120-122 (2000).

25. Sifakis, S., Koukou, Z. & Spandidos, D.A. Cell-free fetal DNA and pregnancy-related complications (Review). Mol Med Rep. 11:2367-2372 (2015).

26. Alberry, M.S., et al. Quantification of cell free fetal DNA in maternal plasma in normal pregnancies and in pregnancies with placental dysfunction. Am J Obstet Gynecol. 200:98.e1-6 (2009).

27. Al-Nakib, M., et al. Total and fetal cell-free DNA analysis in maternal blood as markers of placental insufficiency in intrauterine growth restriction. Fetal Diagn Ther. 26:24-28 (2009).

28. Smid, M., et al. Correlation of fetal DNA levels in maternal plasma with Doppler status in pathological pregnancies. Prenat Diagn. 26:785-790 (2006).

29. Thornburg, K.L. & Louey, S. Uteroplacental circulation and fetal vascular function and development. Curr Vasc. 11:748-757 (2013).

30. Haeussner, E., Schmitz, C., Frank, H.G. & Edler von Koch, F. Novel 3D light microscopic analysis of IUGR placentas points to a morphological correlate of compensated ischemic placental disease in humans. Sci Rep. 6:24004 (2016).

31. Karimu, A.L. & Burton, G.J. The effects of maternal vascular pressure on the dimensions of the placental capillaries. Br J Obstet Gynaecol. 101:57-63 (1994).

32. Kellow, Z.S. & Feldstein, V.A. Ultrasound of the placenta and umbilical cord: a review. Ultrasound Q27:187-197 (2011).

33. Abramowicz, J.S. & Sheiner, E. Ultrasound of the placenta: a systematic approach. Part II: functional assessment (Doppler). Placenta 29:921-929 (2008).

34. Gomez, O., et al. Sequential changes in uterine artery blood flow pattern between the first and second trimesters of gestation in relation to pregnancy outcome. Ultrasound Obstet Gynecol. 28:802-808 (2006).

35. Brosens, I., Pijnenborg, R., Vercruysse, L. & Romero, R. The "Great Obstetrical Syndromes" are associated with disorders of deep placentation. Am J Obstet Gynecol. 204:193-201 (2011).

36. Adams, T., Yeh, C., Bennett-Kunzier, N. & Kinzler, W.L. Long-term maternal morbidity and mortality associated with ischemic placental disease. Semin Perinatol. 38:146-150 (2014).

37. Palmer, S.K., Zamudio, S., Coffin, C., Parker, S., Stamm, E. & Moore, L.G. Quantitative estimation of human uterine artery blood flow and pelvic blood flow from redistribution in pregnancy. Obstet Gynecol. 80:1000-1006 (1992).

38. Boyd, J.D. Morphology and physiology of the utero-placental circulation. In: Villee CA editor. Gestation. pp. 132-194 (Macy, New York, 1956).

39. Gravemeier, V., Gee, M.W., Kronbichler, M. & Wall, W.A. An algebraic variational multiscale-multigrid method for large eddy simulation of turbulent flow. Comput Methods Appl Mech Engrg. 199:853-864 (2010).

40. Hughes, T.J.R., Scovazzi, G. & Franca, L.P. Multiscale and stabilized methods. In: Stein E, de Borst, R., Hughes, T.J.R. editors. Encyclopedia of Computational Mechanics. pp. 5-59 (John Wiley & Sons, Chichester, 2004).

41. Comerford, A., Gravemeier, V. & Wall, W.A. An algebraic variational multiscale- multigrid method for large-eddy simulation of turbulent pulsatile flows in complex geometries with detailed insight into pulmonary airway flow. Int J Numer Meth Fluids. 71:1207-1225 (2012).

42. Gravemeier, V., Comerford, A., Yoshihara, L., Ismail, M. & Wall, W.A. A novel formulation for Neumann inflow boundary conditions in biomechanics. Int J Numer Meth Biomed Engng. 28:560-573 (2012).

43. Ahrens, J., Geveci, B. & Law, C. ParaView: An End-User Tool for Large Data Visualization (Visualization Handbook Elsevier, 2005).

44. Ayachit, U. The ParaView Guide: A Parallel Visualization Application Kitware (2015).

45. Haeussner, E., Schmitz, C., von Koch, F. & Frank, H.G. Birth Weight correlates with Size but not Shape of the Normal Human Placenta. Placenta 34:574-582 (2013).

46. Roth, C.J., et al. Correlation between alveolar ventilation and electrical properties of lung parenchyma. Physiol Meas. 36:1211-1226 (2015).

47. Harris, J.W.S. & Ramsey, E.M. The morphology of human uteroplacental vasculature. Contrib Embryol. 38:43-58 (1966).

48. Geuzaine, C. & Remacle, J.F. Gmsh: a three-dimensional finite element mesh generator with built-in pre- and post-processing facilities. Int J Numer Methods Eng. 79:1309- 1331 (2009). Link: http://gmsh.info/, Accessed: 14-03-2016.

49. Beck, T. Placental morphometry using a computer assisted measuring programme: reference values for normal pregnancies at term. Arch Gynecol Obstet. 249:135-47 (1991).

50. Lewis, H. Gray’s Anatomy. (Lea & Febiger, Philadelphia, 1920).
